# Influence of different sample preparation techniques on imaging viruses and virus-like particles by scanning electron and scanning transmission electron microscopes

**DOI:** 10.3389/fmicb.2023.1279720

**Published:** 2023-11-14

**Authors:** Monika Kąkol, Ezher Tagliasacchi, Andrzej Borkowski, Mirosław Słowakiewicz

**Affiliations:** ^1^Faculty of Geology, University of Warsaw, Warsaw, Poland; ^2^Faculty of Engineering, Pamukkale University, Denizli, Türkiye; ^3^Faculty of Geology, Geophysics and Environmental Protection, AGH University of Science and Technology, Kraków, Poland

**Keywords:** protocols, bacteriophages, virus-like particles, scanning transmission electron microscopy, scanning electron microscopy

## Abstract

Scanning electron microscopy (SEM) and scanning transmission electron microscopy (STEM) were applied in many laboratories to visualize and image viruses and virus-like particles (VLPs). Two bacteriophages, P1 and Φ6, were chosen as model microorganisms known for their distinct structure, and viruses obtained from biofilms associated with modern travertines (Terme di Saturnia, Italy; Karahayıt “Kızılsu” and Pamukkale, Turkey) were also investigated. Three protocols, (1) *full*, (2) *simplified*, and (3) *all at once* were developed and tested for sample preparation and imaging. The *full* procedure enabled the observation of P1 bacteriophages, whereas the *simplified* protocol, successful in visualizing Φ6, did not yield satisfactory results for P1. The preservation state of the latter appeared to be compromised and led to less informative images in SEM and STEM. Viruses in biofilms exhibited various levels of mineralization and aggregation, complicating their characterization. In the *all at once* procedure, although effective in preserving bacteriophage tails, excessive coating and thickening of samples with heavy chemical reagents led to a reduction in overall image quality. Despite a final washing step, some residues of chemical reagents (OsO_4_ and uranyl acetate) remained, impacting the clarity of the images. Finally, the results obtained emphasize the critical role of sample preparation and imaging techniques in effectively visualizing and characterizing viruses and VLPs. The choice of analytical procedure significantly influences the resolution and preservation state of the observed bacteriophages and VLPs. It is suggested that the appropriate imaging technique is carefully selected based on the specific objectives of the project and the nature of the samples being investigated to obtain the best images of the viruses.

## 1. Introduction

Undoubtedly, viruses play a great role in natural ecosystems. They influence the microbial world through bacterial lysis and horizontal gene transfer (Carreira et al., 2020). An estimated abundance of viruses in the ocean is 10^30^ or between 10^6^ and 10^11^ viruses per milliliter, and similarly, a high number of viral infections occur every second (Suttle, 2007). Despite their important role in the environment, there are still many issues with viruses remaining.

Analysis and structural identification of viruses cannot be revealed without the development of specific microscopic techniques and protocols used to observe objects of 20–300 nm in size. The first electron micrographs of viruses were published by von Borries et al. (1938). They managed to image the outline of cell organelles, and larger viruses magnified up to 20,000 times. Viruses were distorted because samples were only fixed in glycerine (von Borries et al., 1938). Next, Ruska (1940) presented the first micrographs of bacteriophages. He used filtered phages, probably bacteriophage T7 (Ackermann, 2011). The lysate was mixed together with aluminum oxide to adsorb phage proteins. They were described as small round particles, 60 nm in diameter, occurring in aggregates that were easily destroyed by electrons so that only ring-shaped or concave bodies remained.

Nowadays, electron microscopes can achieve a resolution of greater than 0.05 nm, which is 4,000 times better than the light microscope. The sample preparation methods used for applying different microscopic techniques include chemical fixation (using formaldehyde, glutaraldehyde, or osmium tetroxide), cryofixation (with liquid nitrogen or helium), dehydration (by freeze-drying, critical point drying, or with ethanol or acetone), embedding (with resin/epoxy), ultrathin sectioning and polishing, staining (using lead, uranium, or tungsten), freeze-fracturing (sample is cryofixed and later fractured), ion beam milling (in order to make the sample transparent), and finally conductive coating (with an electrically conducting material such as gold, carbon, platinum/palladium, or iridium) (ThermoFisher, 2022). However, the most important problem is the appropriate choice of fixation and staining methods. Paradoxically, the better resolutions and capabilities of electron microscopes often force more careful preparation of samples. A key issue is the need to minimize the possibility of artifacts that can be mistaken for real structures. This issue is particularly crucial when examining environmental samples. In addition, it should be noted that with such a specific material as viruses, this problem becomes extremely important. When looking for the presence of biological particles in clastic material, mineral material, or in the form of biofilms growing on sediment particles, finding bacterial or cyanobacterial cells is difficult. However, searching for particles having smaller orders of magnitude like viral capsids can present a bigger problem. This is more important under the conditions of modern carbonate sedimentary environments, for example, bacteria and viruses probably play an essential role in affecting mineral phase precipitation (Słowakiewicz et al., 2021). Moreover, this issue applies not only to modern sediments but also to ancient carbonate formations, where traces of past biological activity may have been preserved.

The aim of this study is to image mineralized and non-mineralized viruses and virus-like particles (VLPs *sensu* Pacton et al., 2014) and prepare a protocol for the most optimal methods of sample preparation to be used for analyses observed under an electron microscope. In the conducted experiments, pure bacteriophages were selected as the main reference material to test different protocols. It was important to observe the effect of the protocols on the pure and different bacteriophages. Those viruses isolated from biofilms associated with modern travertine sites were prepared only using the *full* procedure, to observe the visual effect of extracting them from their natural environment. Imaging of viruses obtained from environmental and phage lysate samples requires a cautious application of numerous verified protocols. While some of the protocols seem to be appropriate for specific viruses and VLPs, they may not be effective for others. Here, both the scanning electron microscope (SEM) and scanning transmission electron microscope (STEM) have been used as they are accessible instruments in most laboratories. The application of these two instruments revealed the advantages and disadvantages of the methods being tested for the virus and VLP imaging. In this study, three different protocols were applied to image bacteriophages, and one protocol was applied for the natural samples.

## 2. Materials and methods

Two bacteriophages (*Escherichia* phage P1 and *Pseudomonas* phage Φ6) and viruses isolated from three travertine biofilm samples were selected for microscopic analyses. All protocols were applied at room temperature, and the samples were stored in a fridge at 4°C.

### 2.1. Sample collection and preparation

#### 2.1.1. Bacteriophages

Preparation and purification procedures of *Escherichia* phage P1 (DSMZ-5757) and *Pseudomonas* phage Φ6 (DSMZ-21518) have been presented by Działak et al. (2022). In brief, P1 and Φ6 bacteriophages were obtained from the Leibniz Institute DSMZ—German Collection of Microorganisms and Cell Cultures. For propagation of *Escherichia* phage P1 and *Pseudomonas* phage Φ6, *Escherichia coli* (DSMZ-5698) and *Pseudomonas syringae* van Hall (DSM-21482) were used. Bacteriophages were cultured using the modified double-agar layer method (Kropinski et al., 2009). After cultivation, the top-agar layer was placed in a 50 mL polypropylene tube and vigorously shaken with Tris-MgCl_2_ buffer. Then, the tube was centrifuged (4,400 × *g*) to remove all the residues of agar and bacteria. Subsequently, the supernatants were transferred to 2 mL polypropylene tubes and centrifuged (24,250 × *g*). The supernatants were completely discarded, and viral pellets were resuspended in 0.9% NaCl solution and filtered using a syringe filter (RC, 0.22 μm). The bacteriophage yield was set at 10^10^ virions/mL.

#### 2.1.2. Isolation and concentration of viruses from natural samples

Three biofilm samples covering the active depositional surface of carbonate travertines or interlayered with travertine were collected from Terme di Saturnia (42°39′31.23″N, 11°30′59.61″E; Italy), Pamukkale (37°55′24.46″N, 29°07′23.28″E), and Karahayıt “Kızılsu” (37°58′2.42″N, 29°6′9.30″E; Turkey) (Słowakiewicz et al., 2023). In total, 0.1–0.8 g of each biofilm sample, depending on the expected concentration of viruses, was placed in a sterile Eppendorf tube; 2% glutaraldehyde was added to equalize the weight of each Eppendorf tube to 1 g. The samples were subsequently stored in the fridge at 4°C for 15 min. Next, 9 μL of ethylenediaminetetraacetic acid (0.1 mM EDTA) was added to each sample and left for 15 min at 4°C (Carreira et al., 2020). Then, each sample was homogenized by triple sonication (in water at room temperature), lasting for 1 min, and mechanical grinding was carried out with a glass rod in between (see Table 1; Danovaro and Middelboe, 2010).

**TABLE 1:** Comparison of different procedures regarding virus isolation from sediment samples.

References	Chemical treatment	Physical treatment
Carreira et al., 2020	EDTA (0.1 mM) for 15 min on ice Benzonase (1 μL) for 30 min at 37°C	Ultrasonic probe (10 s, 3 times)
Danovaro and Middelboe, 2010	Na_4_O_7_P_2_ (5–10 mM) for 15 min on ice DNase (1 μL) + RNase (1 μL) for 15 min at room temperature	Ultrasonic bath (1 min, 3 times)
Pan et al., 2019		Shake and vortex Ultrasonic bath on ice (1 min)
This study	EDTA 0.1 mM for 15 min in the 4°C	Ultrasonic bath at room temperature water (1 min, 3 times) Mechanical grinding in between

**FIGURE 1:**
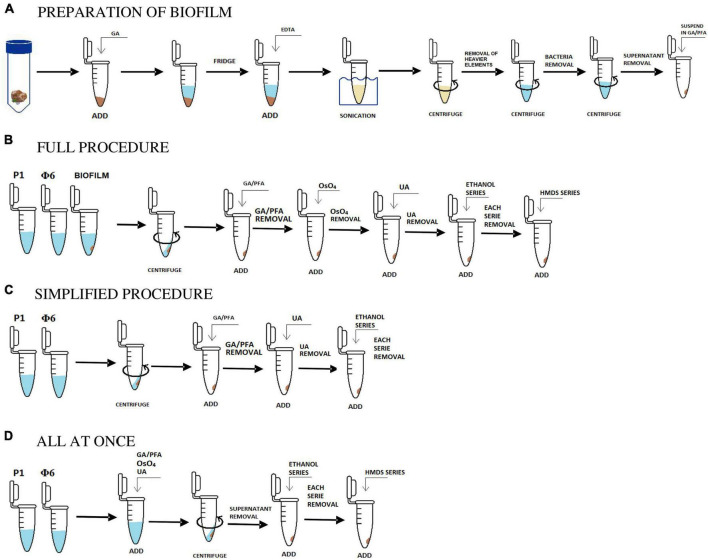
Scheme in **(A)** shows the initial stage of the preparation of biofilm samples and **(B,C,D)** are the applied and tested procedures. Description in sections “2.1. Sample collection and preparation, 2.1.1. Bacteriophages, 2.1.2. Isolation and concentration of viruses from natural samples, and 2.2. Protocols.”

In the next step, the homogenized suspension was centrifuged at 5,000 × *g* for 3 min in order to remove heavier rock particles. Then, the supernatant was placed in a new sterile Eppendorf tube and re-centrifuged at 18,000 × *g* for 3 min. This step was repeated to make sure that most of the bacterial cells were removed. The obtained solution contained only viruses and VLPs and some small clay particles, which could not be removed (Figure 1A).

### 2.2. Protocols

#### 2.2.1. The *full* procedure

The *full* procedure was applied and described for both natural and bacteriophage samples. Two bacteriophage samples (P1 and Φ 6) were prepared as described in section “2.1.1. Bacteriophages.” Three natural samples were initially prepared. The preparation process included the isolation of bacteriophages, viruses, and VLPs from natural samples using sonication, EDTA, and centrifugation as shown in section “2.1.2. Isolation and concentration of viruses from natural samples” and Figure 1A.

Overall, 1 mL of initially prepared biofilm sample and bacteriophage lysates were collected after prior shaking. Each suspension was placed in an Eppendorf tube and centrifuged at 18,600 × *g* for 60 min [based on the procedure proposed by Williams and Fraser (1953)]. The obtained pellet was fixed in a 2.5% mixture of 500 μL glutaraldehyde (GA) and paraformaldehyde (PFA, 1:1, v/v) and stored at 4°C for 24 h (Abay et al., 2019). The supernatant was drawn off the Eppendorf tube, and the pellet was stained with 250 μL osmium tetroxide (4% OsO_4_) and left for 15 min (Palade, 1952). After drawing off the supernatant, the pellet was stained again with 250 μL uranyl acetate (UA, 4%) and left for 15 min (McDonald, 1984).

Water in each sample was gradually exchanged with ethanol (25, 50, 75, and 100% C_2_H_6_O). Each washing step lasted for 1 min. Then, C_2_H_6_O was gradually exchanged with hexamethyldisilazane (HMDS mixed with C_2_H_6_O) in the same way as ethanol, i.e., 25, 50, 75, and 100% HMDS (Figure 1B; Shively and Miller, 2009).

#### 2.2.2. The *simplified* procedure

The *simplified* procedure was applied for Φ6 and P1 phages to test how the lack of some aggressive chemical reagents influences the samples. All steps were carried out in the same way as described in section “2.2.1. The *full* procedure,” excluding the addition of OsO_4_ (staining step) and HMDS (drying step). The last step was to gradually exchange water with ethanol and leave it for a few hours to dry (Figure 1C).

#### 2.2.3. The *all at once* procedure

The last tested procedure is called *all at once*, and it was carried out only on P1 and Φ6 phages. This protocol is used for testing how staining with heavy metals influences the centrifugation processes. It included all of the steps from the *full* procedure. However, unlike the *full* procedure, fixation and staining steps were combined, which means that glutaraldehyde/paraformaldehyde, OsO_4_, and uranyl acetate were added *all at once* (Figure 1D). The result showed that the pellet was obtained after 15 min of centrifugation at 18,600 × *g*. Water in the sample was gradually replaced with ethanol and HMDS as applied in the *full* procedure (section “2.2.1. The *full* procedure”).

### 2.3. SEM/STEM investigation

After the preparation of all samples using the three different protocols, 25 μL of each sample was placed on the carbon film-coated transmission electron microscope (TEM) grids and left to dry. The prepared carbon grids were coated with 8 nm of iridium in the Leica Sputter Coater ACE600. Most of the SEM and STEM images were acquired using an FEI Quanta 3D FEG scanning electron microscope with an EDS detector (EDAX Octane Elect EDS System), operating at 30 kV at a magnitude of 30,000×–500,000× and a working distance of 10.2–10.7 mm. The study was completed on a ZEISS AURIGA 60 scanning electron microscope, operating at 30 kV and a working distance of 3.5 mm. The observations were carried out mainly using a secondary electron detector (SE), with bright field (BF), dark-field (DF), and high-angle annular dark-field (HAADF) modes.

## 3. Results

**FIGURE 2:**
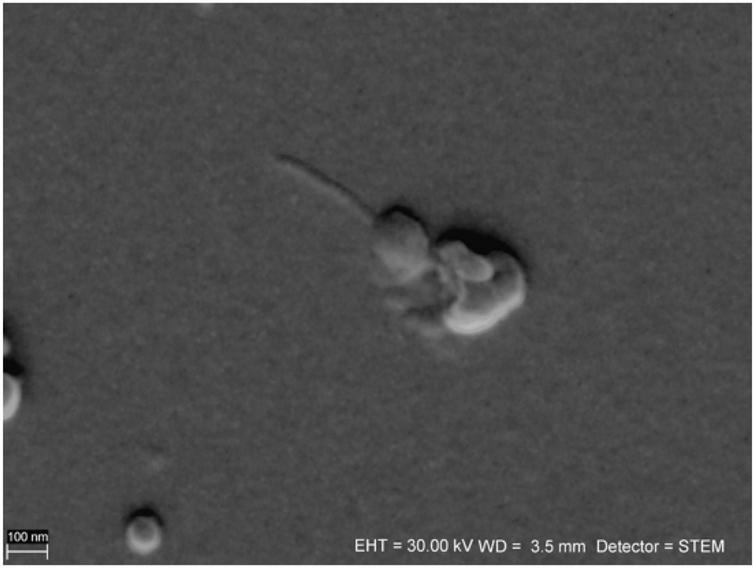
Sample of bacteriophage P1 prepared using the *full* procedure. The head and tail are preserved. The image is taken under ZEISS AURIGA 60 STEM BF mode.

**FIGURE 3:**
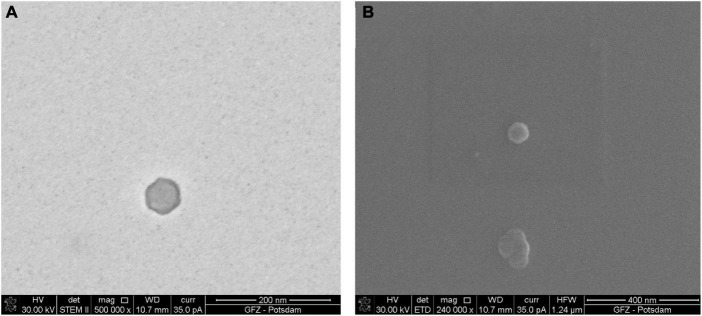
Sample of bacteriophage P1 prepared using the *full* procedure. Comparison of images taken under STEM and SEM BF: **(A)** electrons transmitted through the sample in STEM emphasize the viral capsid shape and the difference in density between the capsid and the inner part of the virus; **(B)** the secondary electrons in SEM generate the topographical contrast causing an effect of a spatial image. As a result of the edge glow effect, viruses under low magnifications are better visible than under STEM.

Although the *full* procedure seems to be the most meticulous and it worked for bacteriophage P1, bacteriophage Φ6 is not observed. The *full* procedure enables observation of the bacteriophages of a varied size. Figure 2 shows an icosahedral head of phage P1 with a tail. Figures 3A, B presents an icosahedral head of phage P1 with a diameter of ∼57 nm.

The images of the smallest VLPs (∼38.5 nm in diameter) are hard to capture because they burn easily under 30 kV accelerating voltage. The image taken under STEM (Figure 3A) shows a sharp viral capsid shape. The resolution is not high enough to observe an inner protein structure, but it enables a border to be distinguished between the two different parts of the virus. The lower resolution image taken under SEM (Figure 3B) shows the surface of the same virus.

The borderline between the capsid and the inner part cannot be easily distinguished, but it is still visible. The images from STEM and SEM complement each other. However, it is possible to define the viral particles using SEM alone.

**FIGURE 4:**
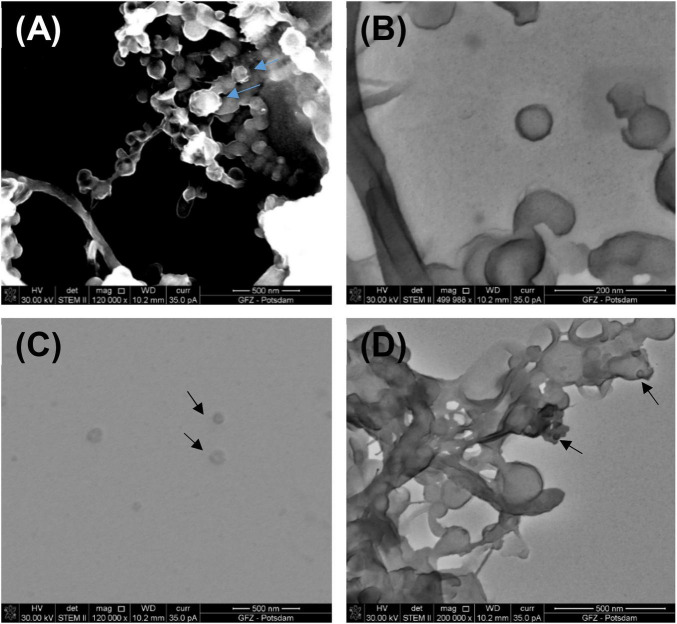
**(A)** Partly mineralized VLPs (indicated by blue arrows) obtained from a living biofilm growing on the fresh calcium carbonate substrate being precipitated from Ca-supersaturated thermal water at Terme di Saturnia. Image taken under STEM HAADF; **(B)** VLPs under STEM BF from the same sample; **(C)** VLPs (black arrows) from Karahayıt “Kızılsu” travertine. Image taken under STEM BF; **(D)** Possible VLPs (black arrows) from Pamukkale travertine. Image taken under STEM BF.

Figure 4 presents images of VLPs obtained from biofilms collected from Terme di Saturnia, Karahayıt “Kızılsu,” and Pamukkale travertines. Here, VLPs are distinctly different from bacteriophages. Partially mineralized and aggregated VLPs are presented in Figure 4A. After image enlargement (Figure 4B), an unspecified VLP with an outline of ∼65 nm can be observed. Around the particle, there is an envelope-like outline, which can be a viral envelope or an effect of partial mineralization.

VLPs in the biofilm samples from Karahayıt “Kızılsu” and Pamukkale (Figures 4C, D) are likely not well preserved. In the Karahayıt “Kızılsu” sample (Figure 4C), there are non-geometric probable VLPs of ∼78 nm in diameter. It is very difficult to determine their shape. In the Pamukkale sample (Figure 4D), VLP shapes are visible but they are much smaller than those from the Terme di Saturnia sample and more difficult to define (Figure 4A).

**FIGURE 5:**
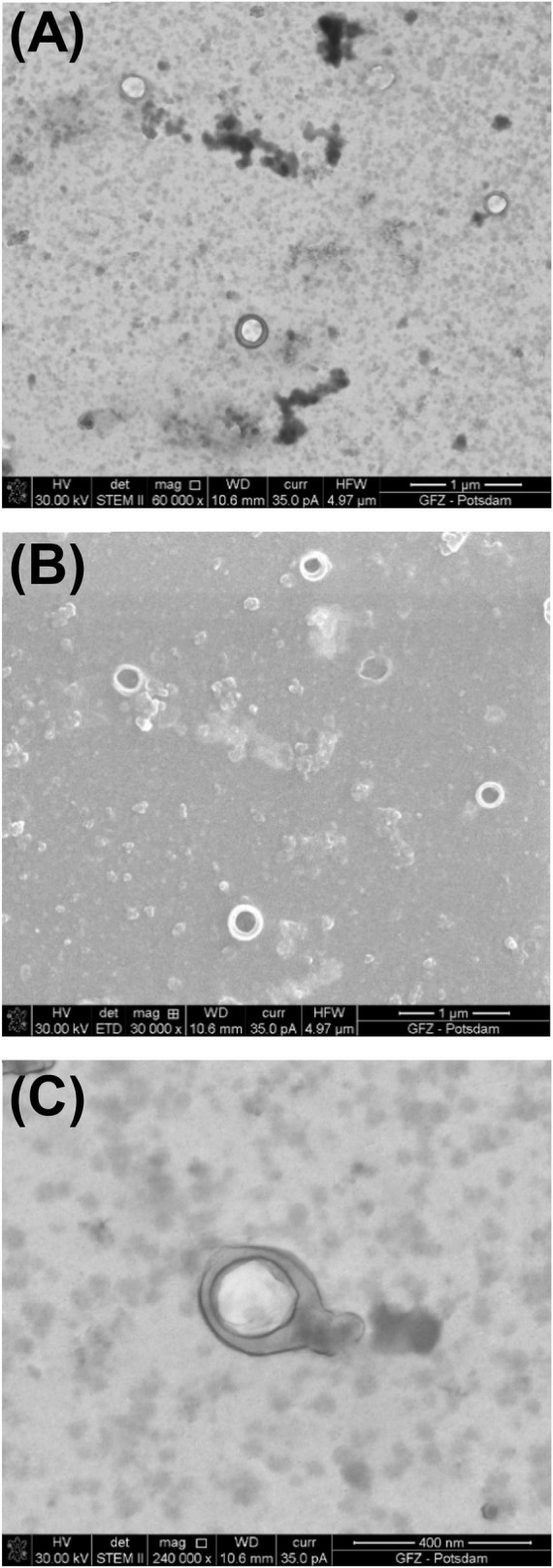
Bacteriophage Φ6 prepared using the *simplified* procedure. **(A)** Image taken under STEM BF; **(B)** image taken under SEM; **(C)** STEM BF image under high magnification shows well-preserved envelope and icosahedral capsid.

P1 and Φ6 are both observed in the *simplified* procedure. In fact, Φ6 is visible including its envelope and capsid. In Figure 5, Φ6 particles have a capsid diameter of ∼200 nm. Here, the P1 particles are poorly preserved.

In the SEM image, the numerous Φ6 envelope’s inequalities on its surface create a characteristic edge effect caused by electrons reflected from the sample surface (Figure 5B). The effect is much less intense on the P1 surface (Figure 3B). This finding can be helpful with surface analysis of particular VLPs.

**FIGURE 6:**
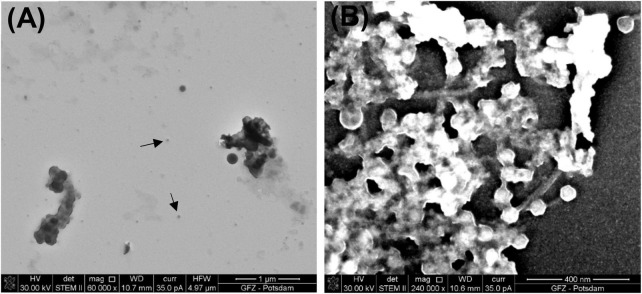
Images of bacteriophage P1. **(A)** Black arrows point to bacteriophage P1 prepared using the *simplified* procedure (STEM BF); **(B)** sample prepared using the *all at once* procedure (STEM HAADF).

The results of the *simplified* procedure differ from the results of the *full* procedure not only in the preservation state of viruses and VLPs but also in the image quality (Figure 6A). The lack of OsO_4_ decreases the contrast of an image during SEM/STEM examination. Moreover, the residue of uranyl acetate is present as a result of preparation without HMDS.

The *all at once* procedure also reveals some difficulties because viruses are coated and glued with heavy chemical reagents, which makes the samples too thick for the acceleration voltage available in STEM (30 kV). However, the higher density of the chemical reagents shortens the centrifugation time and helps to preserve the tails of bacteriophage P1. As a result of this procedure, multiple P1 heads of a similar size (∼67 nm in diameter) are present. Some of them are still connected to their tails (Figure 6B). Although the same final washing step as in the *full* procedure was applied (water replaced with ethanol and then HMDS), a residue of the heavy chemical reagents, such as OsO_4_ and uranyl acetate, is present and glows intensely in the STEM image.

## 4. Discussion

In the present study, three protocols were used for microscopic imaging of mineralized and non-mineralized viruses and VLPs. The steps of the described protocols were tested in different configuration for pure phages and VLPs and viruses extracted from biofilms associated with three modern travertine sites.

All procedures reveal both advantages and disadvantages. The *full* and *simplified* procedures can be used interchangeably, depending on the expected results and available chemical reagents. The *full* procedure provides good contrast, which is better than the *simplified* procedure. However, in both cases, contrast is satisfactory for the SEM/STEM imaging. Interestingly, the *full* procedure turned out to be better for the P1 preservation. The difference between P1 and Φ6 is that P1 has a tail, which is characteristic for such bacteriophages, and has no lipid envelope. The *simplified* procedure seems to be more suitable for bacteriophages with an envelope such as Φ6. This may be caused by the lack of aggressive reagents that could have negatively affected the Φ6 envelope. The use of OsO_4_ in the *full* procedure strengthens the viral structure, while HMDS ensures that the sample will be well dried. In the case of the *full* procedure, metal-containing reagents can cause a harmful effect on some viruses originating from the samples. Using the *simplified* method, the viral structure is not properly strengthened; the P1 tail is not preserved, and it is common to have a uranyl acetate residue that is not fully washed out by ethanol alone.

The application of the *full* procedure to the biofilm sample shows satisfactory results. It enabled the recognition of partially mineralized virus-like particles. The *simplified* procedure has not yet been tested on biofilm samples. This experiment is planned for a future study.

The *all at once* procedure is an interesting example of the impact of combining chemical reagents on the viral pellet and increasing the density of the viral suspension. Obtaining a viral pellet from natural samples is sometimes challenging because the biofilm may contain more or fewer viruses depending on the sample. It is interesting to observe viruses clustered in aggregates. Obviously, this is not a method that can be used for identifying viral material but it can be a tool for observing the presence of viruses and to get a satisfactory viral pellet in the more difficult cases.

**FIGURE 7:**
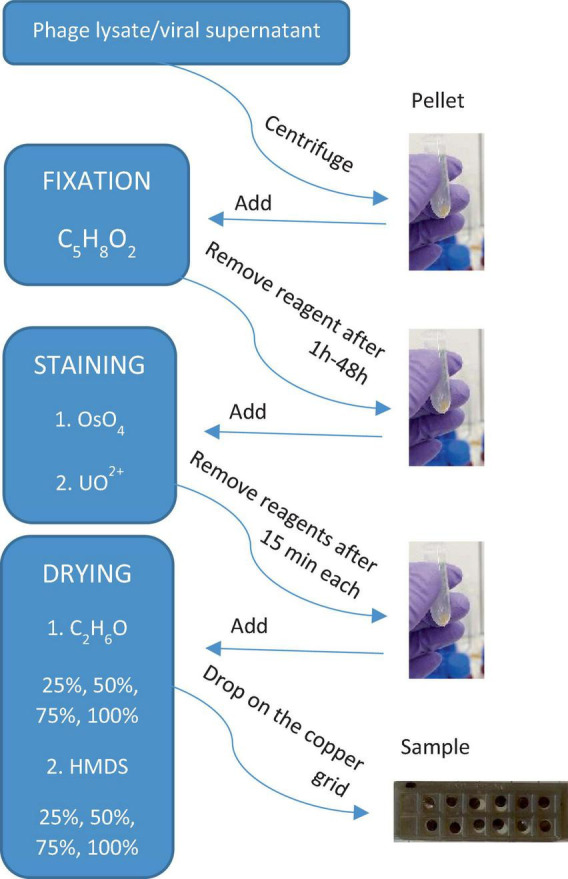
A simple flowchart illustrating the main steps of sample preparation for electron microscopy imaging of viruses and VLPs obtained from phage lysate or viral supernatant (biofilm samples).

The results of the applied procedures are illustrated in a flowchart, presenting the main steps of sample preparation for electron microscopy imaging of viruses and VLPs (Figure 7). Here, it was important to test as many virus samples as possible to get an idea of any possible adverse outcome of certain reagents used for microscopic sample preparation. This study is based on the observations of bacteriophages P1 and Φ6, which have been chosen as representative examples to record the effect of particular chemical reagents on the different protein structures of viruses. Each step is very important and differently influences the final image. For example, fixation starts right after collecting the sample from the natural environment. The most common fixation reagent is glutaraldehyde, which, opposite to OsO_4_, does not kill a microorganism but builds itself into its structure. A comparison of the different biological samples by electron microscopy preparation techniques is presented in Supplementary Table 1.

Staining is necessary for the imaging of biological samples under an electron beam (Dykstra and Reuss, 2003), and Table 2 shows the reagents that can be used in this study. Not all of the stains are readily available, and it is essential to understand how the use of a slightly staining reagent, such as uranyl nitrate, instead of OsO_4_, affects the sample preparation. The last step is sample dehydration and drying. These steps are crucial for the quality of imaging so that the sample does not burn under the electron beam. The dehydration step must always be carried out, whereas drying, such as freeze-drying, or the use of a critical point dryer, HMDS or TMS, as advanced methods and not commonly available drying agents, can be neglected at the expense of image quality (Dykstra and Reuss, 2003).

**TABLE 2:** Chemical reagents used in electron microscopy biological sample preparation based on the study by Reynolds (1963), Dykstra and Reuss (2003), McCutcheon and Southam (2018), and Abay et al. (2019).

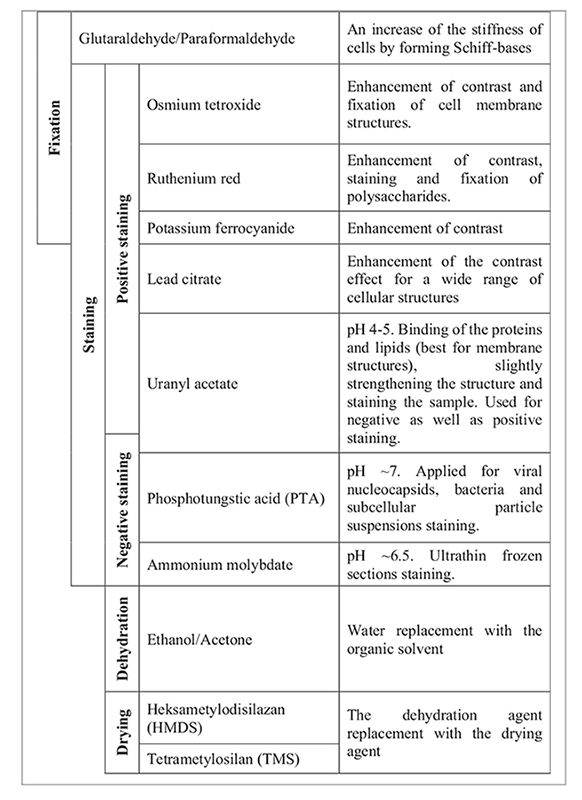

### 4.1. Choice of chemical reagents

Here, no common buffer, such as the popular NaCl, cacodylate buffer (McDonald, 1984), or phosphate-buffered saline (McCutcheon and Southam, 2018), was used (Supplementary Table 1). Instead, ultrapure or Milli-Q water was replaced with distilled water, which successfully prevented viruses from a possible burst. All samples were stained in the acidic pH using uranyl acetate. It was important to decide whether the pH of the reagent used for staining should be acidic or basic. Nermut (1982) reported that acidic pH helps to observe membrane structures (such as envelopes of viruses), and a basic pH is better for internal structures such as nucleocapsids. Certainly, the envelope of Φ6 is clearly shown in the obtained images, whereas P1 is a little more difficult to define.

In biological sample preparation, glutaraldehyde mixed with formaldehyde is commonly used as the first chemical reagent for fixing the structure of the examined viruses. It strengthens the structure of the virus even before it is prepared. In addition to glutaraldehyde, uranyl acetate and OsO_4_ also have a fixing effect (Palade, 1952). In some procedures, the staining and fixing stages are combined (Supplementary Table 1). These two stages were combined in the *all at once* procedure, in order to check the difference in the OsO_4_ and uranyl acetate interaction, which were added simultaneously with the fixing agent. McCutcheon and Southam (2018) used a combination of glutaraldehyde, paraformaldehyde, and ruthenium red.

The next but *very important* stage is the proper staining of the sample. For that, uranyl acetate and OsO_4_ can be applied. The intensity of the sample’s contrast observed under the electron microscope depends on the atomic weight of the stain attached to the structure of the examined viruses. The most efficient staining can be obtained when not one but double stains are used for contrast (e.g., McDonald, 1984). McCutcheon and Southam (2018) in their experiments concluded that uranyl acetate is important for preserving cell structure when it was additionally applied in one procedure (Supplementary Table 1). Here, no proper comparison is mentioned in this case, as uranyl acetate is used in every method. A comparison with and without the use of OsO_4_ was applied herein, which is more widely used for solution staining than negative staining (Palade, 1952; McDonald, 1984), due to its poisonous nature and very complicated negative staining preparation techniques (Barland and Rojkind, 1966).

Next, it is also very important to wash the samples properly. Here, washing was tested with ethanol. Various ethanol dilution series have been described (Supplementary Table 1). For the purpose of this study, a *simplified* procedure (25, 50, 75, and 100% C_2_H_6_O) was used due to the risk of washing out and losing the viral pellet. However, in some cases, it is better to repeat the washing step, i.e., in 100% ethanol (McCutcheon and Southam, 2018) or 90% acetone (McDonald, 1984), even several times. Samples dehydrated in ethanol or acetone can be later dried in the critical point dryer.

### 4.2. Centrifugation force

Herein, different centrifugation forces were tested. The best centrifugation condition is centrifugation in a lower *g*-force (18,600 × *g*) for a longer time (60 min) to obtain a visible pellet. In the *all at once* procedure, the centrifugation time was four times shorter compared with the other procedures. This was because of the higher density of the reagents in the suspension. The centrifugation process is crucial for the P1 tail preservation. The tails were sometimes preserved in the *full* procedure (Figure 2). However, Williams and Fraser (1953) suggested that T-bacteriophages were losing their tails at 65,000 × *g*. The maximum centrifugation force used herein was 18,600 × *g*, which does not exceed the mentioned value.

### 4.3. Mineralized, non-mineralized VLPs and viruses, and clay particles

Viruses from the biofilm samples were successfully extracted using EDTA and sonication. EDTA was added at the beginning to start the detachment process from the chemical treatment. It was used to release viruses associated with extracellular polymeric substances (EPS) and remove present cellular membrane lipids (Table 1; Carreira et al., 2020). The problem that may occur is that clay minerals which are lighter than the other particles and fall down at the same time as viruses during centrifugation.

After some time from when the obtained viral pellet has been put aside, the solution usually changes color. This is due to the presence of clay minerals. This factor is problematic to eliminate because these particles can be as light as viruses. To eliminate the presence of these particles as effectively as possible, filtering the solution after centrifugation of bacterial particles can be applied (Feng et al., 2023). After the last step of centrifugation, the pellet can be left until the color of the supernatant changes, and then the supernatant is removed together with the dispersed clay particles. It turned out that a density separation method can also be efficient for the separation of virus and VLP from the sediment (Pan et al., 2019).

Biofilm samples may arouse controversy because it is commonly assumed that the biofilm contains mineralized or partially mineralized viruses and that they occur often in agglomerates (Pradhan et al., 2022) or are included in the EPS (Słowakiewicz et al., 2023). Distinguishing nanometer-sized viruses from other mineral particles still causes problems mainly because a compositional analysis may be distorted by a mineral growing on the virus. Therefore, in the case of imaging viruses and VLPs under an electron microscope, other identifying methods should be applied. Nowadays, the shape and size of the particle seem not to be diagnostic enough; even if in some cases, it may be exceptionally distinctive and very unlikely for the other particles to occur in this form (Figure 5), but also other experimental methods can be applied, such as the carbon/nitrogen/phosphorus (C/N/P) ratio measurement within the microorganism community (Jover et al., 2014).

### 4.4. Microscope disruptions

During electron microscopic examination, numerous disruptions have to be considered, such as lens aberration, electron charge, edge effect, or sample burning (Schatten and Pawley, 2008). Lens aberration is an effect caused by the longer electron wavelengths and can be corrected by adjusting the aperture (Schatten and Pawley, 2008). Lens aberration causes a blurred image. Since it is operated with magnifications of ∼200 nm, lens aberration often occurs, and not only the aperture size but also the frequent adjustment of the wobbler should be adjusted.

The size of the aperture varies in different microscopes.

During imaging, the user must decide on a case-by-case basis whether to use high current or not. Switching the high current on commonly provides a better-quality image but only on the condition that the sample is very well dried and does not burn drastically. In some cases, it may also cause intense electrification of the sample.

An electron accelerating voltage of 30 kV is suitable for a very well-dried specimen. The burning of the sample was not troublesome in the *full* procedure. Unfortunately, in the case of the *simplified* procedure and sometimes in the *all at once* procedure, the analyzed sample did burn, which often made it impossible to set the proper focus of the microscope. The burning could be caused by the residual water or the staining reagents not being well rinsed out.

## 5. Conclusion

The present study has shown differences in the sample preparation for electron microscope applicable to both SEM and STEM. These procedures can be successfully used in life sciences to visualize viruses from natural samples and pure bacteriophages.

The applied protocols reveal that it is possible to image and distinguish between viruses and VLPs clearly, even if the resolution of the scanning electron microscope is significantly lower than that of the transmission electron microscope. P1 and Φ6 bacteriophages used in the experiments proved the influence of different reagents on the different structures of viral proteins and allowed the exclusion of the negative influence of the reagents on at least some of the viruses present in the biofilm samples. Each procedure can be applied in virus studies or modified depending on the expected effects and the research needs.

The applied methods provided good preservation of most viruses, a visible difference in the use and non-use of toxic chemicals, and a lack of chemical residues usually visible in the images (in the *full* procedure). Future studies should include testing of more chemical reagents, e.g., different pH, and using other bacteriophages to visualize more differences in the virus preservation.

Finally, it is recommended not to use ultrapure water for the preparation of reagents. Instead, the reagents can be diluted with distilled water. This helps to prevent the sample from tearing without adding additional reagents such as NaCl. The use of toxic reagents, such as HMDS or OsO_4_, is not necessary during sample preparation, but in most cases, the quality of images obtained during SEM/STEM investigation is much higher. Instead of HMDS, the chemical point dryer can be used. Considering the fact that the *full* procedure turned out to be destructive for phage Φ6, for natural samples, the more *simplified* procedure is better. Nonetheless, the *all at once* procedure should be still a subject of study because of its potential to preserve the tails of phages.

## Data availability statement

The original contributions presented in this study are included in this article/Supplementary material, further inquiries can be directed to the corresponding authors.

## Author contributions

MK: Formal analysis, Investigation, Methodology, Writing – original draft. ET: Resources, Writing – review and editing. AB: Writing – review and editing. MS: Writing – review and editing, Conceptualization, Funding acquisition, Supervision.
