# Supplementary material for: Influence of different sample preparation techniques on imaging viruses and virus-like particles by scanning electron and scanning transmission electron microscopes
Source: Front Microbiol. 2023 Nov 14;14:1279720. doi: 10.3389/fmicb.2023.1279720 (PMC10682772; doi:10.3389/fmicb.2023.1279720)
Supplement: Supplementary file 1 [file Data_Sheet_1.docx]

**Influence of different sample preparation techniques on imaging viruses and virus-like particles by scanning electron and scanning transmission electron microscopes**

Monika Kąkol^1*^, Ezher Tagliasacchi^2^, Andrzej Borkowski^3^, Mirosław Słowakiewicz^1*^

^1^ Faculty of Geology, University of Warsaw, ul. Żwirki i Wigury 93, 02-089 Warsaw, Poland

^2^ Faculty of Engineering, Pamukkale University, Kınıklı Campus, 20160 Denizli, Turkey

^3^ Faculty of Geology, Geophysics and Environmental Protection, AGH University of Science and Technology, Al. Mickiewicza 30, 30-059 Kraków, Poland

Corresponding authors: [m.kakol3@student.uw.edu.pl](mailto:m.kakol3@student.uw.edu.pl); [m.slowakiewicz@uw.edu.pl](mailto:m.slowakiewicz@uw.edu.pl); [m.slowakiewicz@gmail.com](mailto:m.slowakiewicz@gmail.com)

| Stage/Reference | McCutcheon and Southam (2018) | | | McDonald, (1984) | Palade, (1952) | Feng et al., (2023) | This study | | |
| --- | --- | --- | --- | --- | --- | --- | --- | --- | --- |
|  | 1 | 2 | 3 |  |  |  | Full | Simplified | All at once |
| Pre-fixation |  |  |  |  |  |  | 2% glutaraldehyde | 2% glutaraldehyde | 2% glutaraldehyde |
| Centrifugation/  filtration |  |  |  |  |  | 6,500×g for 15min at 15°C | 5,000×g for 3 min | 5,000×g for 3 min | 5,000×g for 3 min |
|  |  |  |  |  |  | filtered through a 0.22-μm filter | 18,000×g for 3 min | 18,000×g for 3 min | 18,000×g for 3 min |
|  |  |  |  |  |  | pellet - centrifugation at 12,000×g for 30min at 4°C | pellet - 18,600×g for 60 min | pellet - 18,600×g for 60 min | pellet - 18,600×g for 15 min |
| Fixation | high-pressure freezing (HPF) | 2.5% glutaraldehyde and 2.0% paraformaldehyde, 75 mM ʟ-lysine and 0.075%(aq) ruthenium red | 2.5% glutaraldehyde and 2.0% paraformaldehyde, 75 mM ʟ-lysine and 0.075%(aq) ruthenium red | buffer+2% glutaraldehyde for 30 min at 37°C | OsO_4_ in double distilled water or buffer |  | 2% glutaraldehyde and paraformaldehyde | 2% glutaraldehyde and paraformaldehyde | 2% glutaraldehyde and paraformaldehyde, 4% OsO_4_, 4% uranyl acetate |
| Staining | quick freeze substitution |  |  | 0.5% OsO_4_ + K_3_Fe(CN)_6_ for 30 min |  |  | 4% OsO_4_ | 4% uranyl acetate |  |
|  | 1%(aq) OsO_4_, 0.5%(aq) uranyl acetate, and 5% (vol./vol.) water in acetone |  |  |  |  |  |  |  |  |
| Additional staining |  | 2.0%(aq) OsO_4_ (Garland et al., 1975) |  | 2% (aq) uranyl acetate for 60-120 min |  |  | 4% uranyl acetate |  |  |
| Buffer |  | 0.1 M sodium cacodylate buffer | phosphate buffered saline (PBS) | 50 mM cacodylate+5 mM CaCl_2_ | acetate-veronal buffer | 0.1M NaCl, 8 mM MgSO_4_·7H_2_O, 50 mM Tris–HCl, 0.005% (wt/vol) glycerol | non | none | none |
| Postfixation |  |  |  |  |  |  |  |  |  |
| Dehydration | aceton | ethanol series: 20%, 40%, 60%, 80%, 90%, 100%, 100%, 100% | ethanol series: 20%, 40%, 60%, 80%, 90%, 100%, 100%, 100% | acetone series: 25, 50, 75, 90, 95, 100% | ethanol series: 70%, 95%, 100% |  | ethanol: 25%, 50%, 75%, 100% | ethanol: 25%, 50%, 75%, 100% | ethanol: 25%, 50%, 75%, 100% |
| Drying |  |  |  |  |  | 2–5 min and air-dried | HMDS: 25%, 50%, 75%, 100% |  | HMDS: 25%, 50%, 75%, 100% |
| Embedding | Resin | Resin | Resin | Resin | Resin |  |  |  |  |
| Sectioning | Ultrathin Sectioning | Ultrathin Sectioning | Ultrathin Sectioning | Silver sections | microtome |  |  |  |  |
| Poststaining |  | lead citrate (Reynolds, 1963) |  | 5 min in 1% uranyl acetate in 70% methanol, 2 min in lead citrate (Reynolds, 1963) |  | 2% uranyl acetate for 30s |  |  |  |

Table S1. The comparison of different biological samples and EM preparation techniques with the present procedures. Main biological samples and preparation stages on which the present study was based are presented.

**References**

Feng, X., Li, Y., Tian, C., Yang, W., Liu, X., Zhang, C., et al. (2023). Isolation of archaeal viruses with lipid membrane from Tengchong acidic hot springs. *Frontiers in Microbiology* 14, 1134935.

Garland, J. M., Archibald, A. R., and Baddiley, J. (1975). An electron microscopic study of the location of teichoic acid and its contribution to staining reactions in walls of Streptococcus faecalis 8191. *Microbiology* 89, 73–86. doi: 10.1099/00221287-89-1-73.

McCutcheon, J., and Southam, G. (2018). Advanced biofilm staining techniques for TEM and SEM in geomicrobiology: Implications for visualizing EPS architecture, mineral nucleation, and microfossil generation. *Chemical Geology* 498, 115–127. doi: 10.1016/j.chemgeo.2018.09.016.

McDonald, K. (1984). Osmium ferricyanide fixation improves microfilament preservation and membrane visualization in a variety of animal cell types. *Journal of Ultrastructure Research* 86, 107–118. doi: 10.1016/S0022-5320(84)80051-9.

Palade, G. E. (1952). A study of fixation for electron microscopy. *Journal of Experimental Medicine* 95, 285–298. doi: 10.1084/jem.95.3.285.

Reynolds, E. S. (1963). The use of lead citrate at high pH as an electron-opaque stain in electron microscopy. *Journal of Cell Biology* 17, 208–212. doi: 10.1083/jcb.17.1.208.
